# Ten tips to carry out video consultations in nephrology

**DOI:** 10.1093/ckj/sfae287

**Published:** 2024-09-14

**Authors:** Alexander Woywodt, Rebecca E Payne, Brooke M Huuskes, Bartu Hezer

**Affiliations:** Department of Renal Medicine, Lancashire Teaching Hospitals NHS Foundation Trust, Preston, Lancashire, UK; General Practitioner and NIHR In-Practice Fellow, Nuffield Department of Primary Health; Care Sciences, Oxford University, Oxford, UK; Centre of Cardiovascular Biology & Disease Research, Cardiorenal Division, La Trobe Institute for Molecular Science (LIMS), La Trobe University, Melbourne, Australia; Department of Microbiology, Anatomy, Physiology & Pharmacology, School of Agriculture, Biomedicine & Environment, La Trobe University, Melbourne, Australia; Erasmus Medical Centre, Transplant Institute, Rotterdam, the Netherlands

**Keywords:** clinical nephrology, COVID-19, kidney transplantation, telemedicine, video consultation

## Abstract

Video consultations have seen increasing use in nephrology since the COVID-19 pandemic with an aim to address constraints in F2F outpatient capacity and also patients’ concerns around risks of infection when attending healthcare facilities. Nephrologists have learned through experience to use video consultations for providing routine follow up and also for *ad hoc* triage of unwell patients. Advantages of video consultations include convenience, cost savings through avoiding clinic overheads, and reducing the carbon footprint of care. The last is increasingly relevant as nephrologists consider climate change and its implications. Video consultations are not a panacea to overcome challenges in nephrology and risks also exist for example when it comes to redesigning pathways and maintaining access to F2F assessments when required. It is equally important to consider practical aspects such as reimbursement, prescribing, and documentation. Some clinicians may wish to carry out video consultations from home to save time spent commuting but this, too, requires careful thought. Another consideration is the digital divide and support should be provided for patients who are less IT literate or who have no access to the digital world. Patients with special needs such as those with visual or hearing impairment and those with language issues also require consideration. We view video consultations as a developing and growing part of the portfolio of renal care. We see their main role in providing routine follow up to stable and IT literate outpatients, particularly where there is provider continuity and where care is provided across a large geographical area.

## INTRODUCTION

The COVID-19 pandemic acted as a catalyst in most health systems to re-think the provision of care while adapting to increased workload and constraints overall [1, 2]. In some health systems, these developments led to a rapid replacement of a proportion of routine F2F appointments by virtual clinics and to the introduction and rapid growth of video consultations. In the UK, the National Health Service (NHS) procured licences for video consultation software in March 2020 in England and later in Wales, while Scotland had used the technology since 2016. Two months later, this platform had already facilitated 79 000 appointments [3]. Convenience is a key advantage particularly where renal centres serve large geographical areas [4, 5]. Remarkably, many institutions do not offer any video consultations. This may be due to problems with infrastructure or reimbursement or because of other barriers, such as lack of awareness. Here, we aim to provide clinicians with a practical guide for starting video consultations.

## TIP 1: CONSIDER REIMBURSEMENT AND THINK ABOUT INFRASTRUCTURE

Reimbursement is a key factor that will determine success or failure and the situation in the UK is a good example; historically, telephone and video consultations attracted very little reimbursement and it was only when the NHS decided to reimburse video consultations in the same way as face-to-face (F2F) encounters that providers could consider a virtual approach more widely [6]. The cost implications of a switch from F2F to video are worth considering including the cost of upfront investment. Detailed work on this aspect is not yet available from within nephrology, although research in other specialities suggests cost savings and improved productivity [7]. Such a switch will probably involve savings through avoiding overheads for F2F clinics (provision and maintenance of rooms, staff, heating, parking, etc.) [8]. Another potential advantage in terms of cost is the ability to start a consultation almost immediately, i.e. without waiting for observations such as weight or blood pressure so that at least in theory more patients can be seen during a session. Additional savings for society involve avoiding time taken off work to attend F2F appointments including time taken off by relatives and carers to bring patients to these appointments [8].

**Figure 1:**
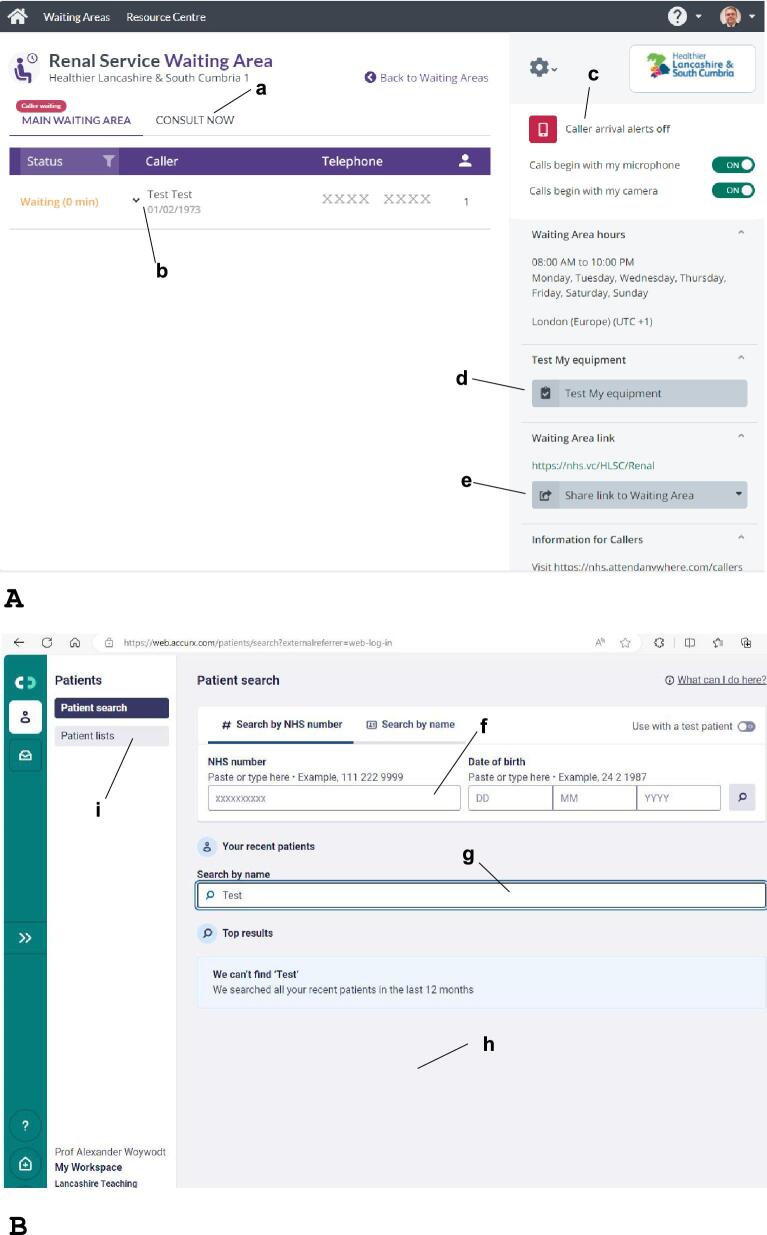
Examples of commonly used video consultation software. (**A**) Attend Anywhere™ (Induction Healthcare, UK). The screen shot shows the virtual waiting area for nephrology video consultations at Lancashire Teaching Hospitals NHS FT, UK. a, Consult now option for making contact outside scheduled clinics whereby the system sends a single use link to the consultation. b, Clicking here opens a window to send a message to this waiting patient, such as ‘expected wait 10 min’. c, Caller alert: the system can be set to send a text message to the provider once a patient enters the virtual waiting room. d, Equipment test: this tests audio, camera, and connectivity. e, Share link function: the provider can send a link to the waiting area via email or text. (**B**) AccuRx (AccuRx Ltd, UK). The main difference in comparison to Attend Anywhere™ is that there is no virtual waiting area. Instead, patients are contacted individually via text. f, Search function for patients in the linked primary care EHR (the link will ensure all demographics are available for contact). g, The search function in the patients recently seen through AccuRx. h, This panel would contain a list of patients seen recently. i, List of recently seen patients by location.

It is also important to think through the implications for infrastructure, to find out whether existing hardware will allow for video consultations, and decide which software to use (Fig. 1). It is also important to be clear about how providers will contact patients i.e. whether the software is linked to an electronic health record (EHR) or whether contact details (email, mobile phone number) will have to be copied from an EHR manually. Legal and regulatory arrangements should be considered as well, this especially around data protection. In the European Union, this will be the General Data Protection Regulation (GDPR). Similar regulation exists in most developed countries. Dedicated video consultation software is usually fully GDPR compliant. It is worth checking the institution's regulations, too, and check whether consent is required. Table 1 sums up five questions clinicians should ask before doing their first video consultation.

**Table 1:** Five questions to ask before doing the first video consultation.

How are video consultations reimbursed compared to F2F appointments?
Can I do video consultations from my office/clinic room and does the hardware allow for video consultations (camera/microphone)?
Which software will be used for video consultations and how will this be funded?
Where are patients' mobile phone numbers and email addresses documented in the EHR and how will the patients be invited i.e. via text message/email invite or written instructions?
Is the proposed setup compliant with local, regional, and national information governance and legal regulations, and is consent required?

## TIP 2: CHOOSE A SUITABLE PATIENT POPULATION AND ENSURE AVAILABILITY OF FACE-TO-FACE ASSESSMENT WHEN REQUIRED

When starting video consultations, providers should consider integration into clinical pathways early on and choose a suitable population of patients, for example transplant recipients or those on home dialysis. These groups of patients tend to have above-average levels of IT literacy and many of them work, often full time. Patients on the transplant waiting list may also warrant consideration as they are, for obvious reasons, usually contactable via smartphone at short notice. Geography may also play a part, and in a regional renal centre, the patients living furthest away may be particularly suitable.

Internet connectivity and locality are also worth considering although rural post codes should not automatically raise concern. In the UK, for example, many remote areas have benefited from government grants for ultra-fast broadband whereas some urban locations have less coverage. In Australia, however, rural broadband signal is often poor. Local knowledge will be key, and patients will usually know whether they have good internet connectivity or not. In our practice, network issues do occur but it these are uncommon, often temporary, and it is unusual for them to preclude a consultation. Many of our younger transplant patients attend video consultations from a confidential area at work, such as an office or staff kitchen that tend to have good internet connectivity.

Stable outpatients are best suited to the beginner. Providers also need to consider how they will provide F2F assessment when required, for example when a patient is unexpectedly unwell. It is also worth thinking through how prescriptions will be done for patients who live far away and how results of laboratory tests and imaging can be assessed.

## TIP 3: CONSIDER TECHNOLOGY FOR HOME MONITORING AND WEARABLES

**Figure 2:**
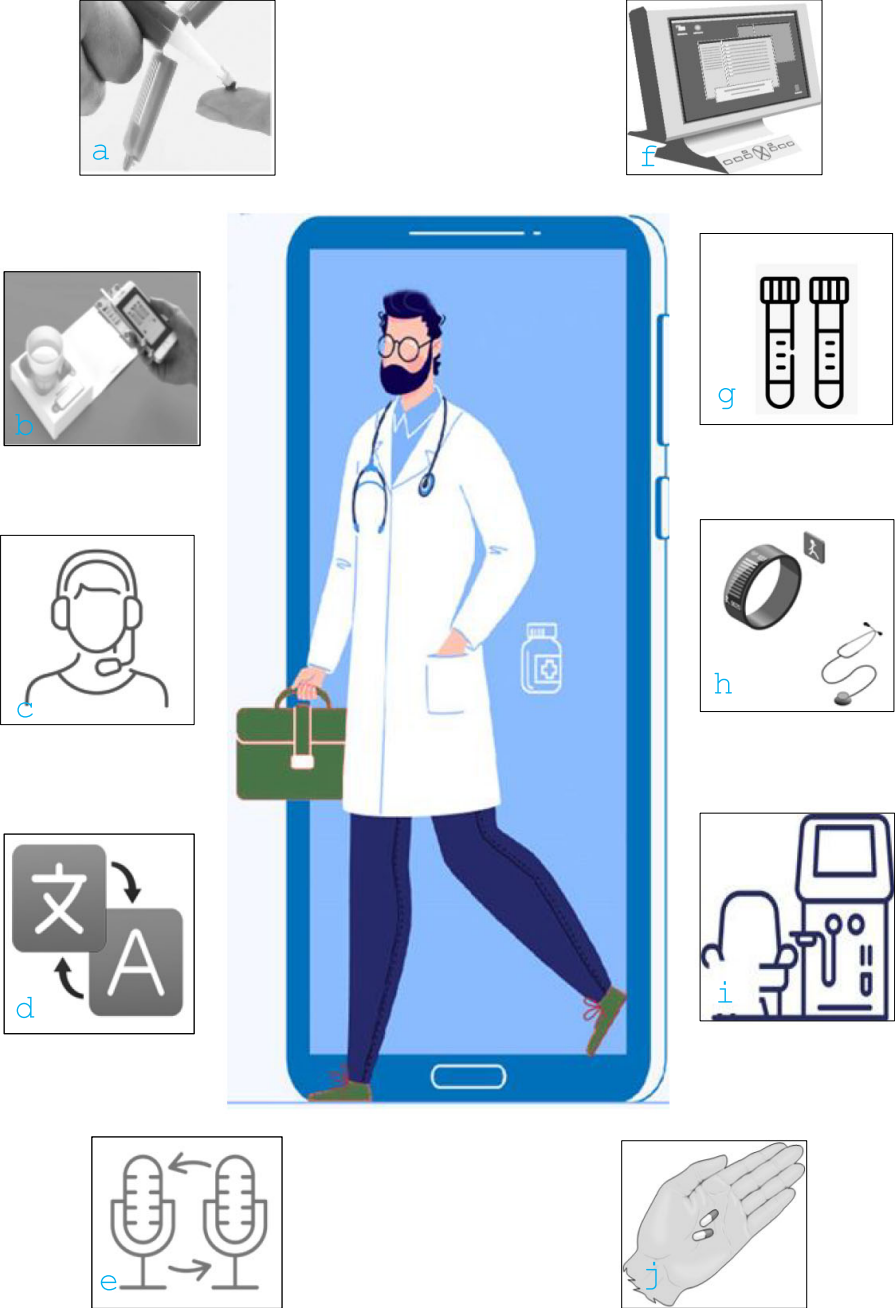
Technology around video consultations: Technology that is already available: (**a**) blood test sampling at home for monitoring of serum creatinine and tacrolimus in transplant patients; (**b**) smartphone-enabled urine dipstick; (**c**) option to let a second person join the video call (for example, psychologist, specialist nurse, colleague, relative etc.); (**d**) option to book an interpreter for the video call; and (**e**) facility for electronic dialogue with general practice (for example, suggesting change in medication, discussion etc.). Future technology: (**f**) full integration with EHR and use of artificial intelligence; (**g**) full portfolio of laboratory tests via home sampling of capillary blood tests; (**h**) integration with wearable technology and enhanced diagnostic functions such as estimating haemoglobin through remote scanning of conjunctival blood vessels [41]; (**i**) integration with dialysis technology; and (**j**) link to vending machines for medication. Symbols obtained from Vectorstock Ltd through commercial licence in July 2024. (Central pictogram, **c**-**i**; **a,b,j** in the public domain.)

Linking video consultation platforms to home-self measurement kit and to wearables [9] is an obvious consideration in the context of video consultations (Fig. 2). Such an approach would encourage patients to self-monitor parameters at home and provide additional data to the medical team. A blood pressure monitor, thermometer, scale, and other (future) wearable devices [10] could be provided in the form of a kit. Remote urinalysis [11] and point-of-care measurement of tacrolimus and creatinine at home are already available [12]. A key challenge will be around the integration of devices into the EHR/video consultation platform; it is simply impractical to use numerous IT platforms concurrently as they all require their own access credentials and compatibility issues also tend to arise when several platforms are required during a video clinic. The issue is particularly relevant where physicians see patients from a large geographical area with a multitude of EHR systems. Full integration into EHR would also facilitate access to patients contact details and clinic lists. Disadvantages of full integration into one monolithic EHR system also exist, mainly around resilience [13].

Another issue will be to avoid data overload and information will have to be displayed in an easy-to-use way, for example in the form of a dashboard. Artificial intelligence could be used to identify patients who need urgent physician input in between scheduled appointments.

Training patients and families to use the kit is probably going to be a key enabler in this approach and dedicated support will have to be available. Maintenance of the equipment is another important issue if the kit is procured through institutions. Points to consider include the cleaning and calibrating as well as repair, and finally replacement of equipment, which may be required periodically. On the other hand, asking patients to buy equipment themselves raises significant issues regarding equal access and may also cause issues with compatibility. This aspect should be included during the planning phase as it will also contribute to cost.

Clinicians will have to have a significant role in ensuring that these reports are reliable and in promoting the use of self-monitoring overall. It will also be important to use the data in clinics in a meaningful way that adds true value to the consultation and convinces patients and families. Peer support is also going to be beneficial beyond sharing their experiences with clinicians and patient support networks are probably required as well with input from medical and technical staff and sharing of challenges and solutions.

## TIP 4: CONSIDER BACKGROUND AND HARDWARE

Careful consideration should be given to the background and the environment where video consultations are carried out. Dedicated video clinic facilities are still rare but where they exist focus on noise reduction and confidentiality. Most platforms allow a choice of digital backgrounds including one with a branded institutional logo or a blur function. If this is not available, then a neutral physical background can be used. Before starting a video clinic, we recommend carrying out a systems check that typically tests connectivity and hardware and allows the clinician to find a suitable camera position. Eye-level communication is best for communicating with the patient and using two monitors is ideal. Having this setup before your video consultation is important to use most of the time with the patient during a call.

We would not routinely recommend the use of mobile phones simply because visual clues are difficult to pick up for clinicians. However, we do recognize that there may be circumstances where clinicians may decide to use their smartphone, for example to overcome IT issues or while away from their hospital base. If possible, we suggest using a phone holder that can be mounted on a screen to sit a decent height from the table.

Camera and microphone equipment also deserve careful thought. Where provided, cameras built into monitors are often poor quality. Clinicians who plan to use video clinics regularly should either invest in a good quality clip on camera after checking that their institution allows the use of private devices at work. Similarly, inbuilt microphones are often suboptimal and most experienced providers of video consultations prefer a headset, which will also provide excellent protection from external noise. A Bluetooth headset will offer the extra advantage of connection to a mobile phone, which helps when there are technical issues and clinicians decide to convert from video to telephone at short notice.

## TIP 5: CONSIDER TRAINING REQUIREMENTS AND RISK

Clinicians need to be trained to consult effectively via video [14]. Issues such as lag can impair communication [15], and using gestures rather than verbal listening noises avoid inadvertently speaking over the patient [16]. Training must address how to communicate and examine effectively via video, and not focus solely on technicalities [17]. Consideration should be given to training in recognizing safeguarding issues [18]. Training also relates to the situation where patients do not attend a scheduled video consultation. A related issue arises where a patient has not received a remote consultation as expected. The patient left in the waiting room at the end of the clinic is a solid reminder that they have not been seen, which is difficult to replicate in the virtual world. It may be useful to clarify expectations in a patient leaflet, for example whether the clinician is expected to make contact via telephone instead.

Risk can be conceptualized at the clinician, patient, and system level. Clinicians need to consider confidentiality, particularly if consulting in an open-plan office or from home. Clinicians should consider removing family photos from the consulting area and avoid distractions during clinic. Configuring the appointments across an entire department is vital and patients can be exposed to harm where video consultation are the purview of a sole enthusiast within a service. Careful consideration also needs to be given to all processes that wrap round a F2F consultation. This includes not just the availability of F2F assessment but also access to further tests such as urinalysis [11] and phlebotomy, for example where patients would usually obtain a blood test by taking a paper form to phlebotomy. It is also important to consider how these processes will be configured where clinicians work off-site [19]. Thought also needs to be given to the provision of information such as information leaflets. Many patients will be able to access information online, but some prefer printed information and processes need to be in place to support this.

## TIP 6: THINK ABOUT WHAT IS EASILY MISSED DURING A VIDEO CONSULTATION

Colours can be distorted on video, making it hard to spot jaundice or cyanosis, especially in patients with darker skin [20]. Skin lesions may be difficult to visualize, this may be resolvable through asking the patient to send a photograph, either via the video software system or through other means [20]. Subtle changes such as increasing frailty or changes in weight are also difficult to recognize via video as patients are typically seated with limited visibility of their lower body. Oedema can be assessed to some degree by asking the patient to point the camera at the ankles but remains difficult to quantify. Managing new shortness of breath is also typically challenging as auscultation and percussion are unavailable during a video consultation [21]. Where a patient is geographically remote from the service, examination, or chest X-ray facilitated by a local general practitioner can complement assessment.

If an illness is not following the anticipated trajectory, or where the clinician feels that clinical examination is required, a patient should be brought in for a F2F encounter. Managing complications of diabetes remotely such as leg ulcers can be challenging and risky. It is wise to regard new diagnoses made over video or telephone as provisional until confirmed by F2F assessment or by investigations [20].

It is also important to acknowledge that non-verbal communication is much more difficult to pick up during video consults than in a F2F setting. Being conscious of posture and body position on camera as well as leaning in towards the camera, nodding and smiling when the patient is talking are some simple things that providers can do to improve non-verbal communication.

## TIP 7: THINK ABOUT THE PATIENT EXPERIENCE

People with chronic kidney disease (CKD) can face a high out-of-pocket health care expenditure. Around 45% of the world's population live in rural settings [22] and those who do are often at a greater socioeconomic disadvantage [23]. Studies have also demonstrated real financial hardship for some patients living with CKD in rural areas, with the biggest expense being travel to access care [24]. Video consultations can reduce this effect by avoiding travel [25]. Not having to pay the associated costs with travelling to a metropolitan centre could mean the difference between paying the electricity bill and having the lights on for some patients. It is worth considering if you really do need to see a patient F2F. Patients often find telehealth efficient, convenient, and easy [26]. Offering telehealth to patients can also reduce anxiety around clinic visits, for example, the stress of driving/getting to a major centre, finding, and paying for a car park, and worrying about potential exposure to contagious pathogens in hospital waiting rooms. Telehealth can also help reduce days taken off work and thereby help to avoid stigma in the work environment. It is also useful to think about how familiar the patient is with the hospital system. Those new to the system might require more support to establish rapport with clinicians. Peer-to-peer patient support is also critical to some patients, and it is important to acknowledge that shared lived experience that can be exchanged in waiting rooms is lost during telehealth. It is important to ask your patient if they would like to be linked in with patient groups and facilitate the connection. Practising cultural safe care in telehealth can profoundly affect health outcomes [27]. It is also important to consider patients who do not speak the primary language and video consultation with an interpreter may be employed [28]. Some software platforms already provide the option to link in a second person such as an interpreter. Similar considerations apply to patients with visual and hearing impairment. Current video consultation software does not feature options for these patients, but progress is highly likely in the near future.

## TIP 8: MAKE VIDEO CONSULTATIONS ENGAGING AND EFFICIENT

Telehealth appointments are most effective when both parties are prepared for the appointment. For the patient, preparation also includes finding a suitably quiet and private space, with no television or radio running and with good internet signal. This is particularly relevant where patients access care while at work. At home, the support of relatives can be extremely useful in some circumstances. Similarly, the clinician should invest in a few minutes of preparation, for example by retrieving relevant laboratory results and reading the most recent clinic letters.

Owing to the chronic nature of kidney disease, patients get to know their nephrologists and look forward to ‘catching up’ at their appointments. Hence, appointments are more than exchanging physiological values between clinician and patient but very much about connection and rapport where we find video consultations vastly superior to telephone encounters. This is particularly relevant where clinicians do video consultations from home and clinicians may want to consider how much of their private lives they want to share.

Clinicians should also have a virtual ‘toolbox’ for technical challenges during the consultation although these are uncommon in our practice. Most video consultation software also has a ‘refresh’ function. Software issues are often related to browser compatibility and IT literate patients will be able to retry on a different browser. Network issues do occur and closing the consultation and re-joining often overcomes the issue. Patients will often know where to access the best signal in their locality. If connectivity is poor video can be deactivated temporarily, which often improves audio signal. Conversion to a telephone consultation may be useful if the consultation is fraught with technical issues. Having a Bluetooth headset that connects to both PC and telephone is ideal because a switch to telephone consultation can occur with minimal delay. On occasion, we demonstrate use of the system during a F2F appointment, for example in patients who are less IT literate. Written instructions for the video clinic are also useful, particularly where relatives or friends facilitate a consultation. Such instructions should also involve a plan B for when a video consultation does not work: i.e. whether the appointment will be rebooked or whether the clinician is expected to phone the patient.

It is also useful to acknowledge that sometimes a lot of important information is exchanged during a consultation and that nephrologists are unable to physically draw diagrams or pass things on to patients during a video consultation. In addition to a traditional clinic letter, it may be useful to share the screen with relevant laboratory results and imaging during the consultation. When sharing, try to ensure that patients can read the information on your screen and use zoom where appropriate. Many software platforms feature chat lines that can be used to share written information or links to patient information websites. Remember that video consults are a two-way system and try to address patients’ questions.

## TIP 9: THINK ABOUT CLIMATE CHANGE AND PROMOTE VIDEO CONSULTATIONS AS AN APPROACH TO REDUCE THE CARBON FOOTPRINT OF CARE

Climate change is now viewed as a key threat to humanity [29] and nephrologists have started considering the carbon footprint of care [30]. Phasing out fossil fuels is a key pillar of achieving targets [31] and converting routine appointments to video consultations can deliver significant reductions in the carbon footprint [25], particularly where care is delivered across a large geographical footprint [32]. It is worthwhile noting that in most institutions video consultations were developed as an approach to overcome constraints of the COVID-19 pandemic and not primarily as a response to climate change. It is therefore important to emphasize this additional benefit [33] not just to colleagues and managers but also towards patients. In our experience, patients and families are often quite open to acknowledging this advantage of video consultations and starting dialogue with patients [34] on this aspect of their care is useful.

## TIP 10: THINK ABOUT THE DIGITAL DIVIDE AND SUPPORT PATIENTS WHO ARE LESS IT LITERATE

A common error is to assume that by now everybody is online [35] and it is possible to lose sight of the digital divide, which describes the gap between those members of society who have full access to digital technologies and those who do not. There is an overlap between digital and social exclusion and poverty. Some patients may volunteer information about their lack of access to the digital world whereas others may not, often due to fear of embarrassment. A 2020 survey of UK bank customers demonstrated that as many 16% of participants were unable to use the internet [36]. It is also important to consider a bigger group of patients who do have smartphone and/or computer and internet access but struggle to access video consultations due to low IT literacy. In our experience, many of those patients can do video consultations with some support, for example by demonstrating the technology during a F2F clinic. Providers and institutions should refrain from labelling such patients as ‘ineligible’ and instead consider support mechanisms, such as a digital navigator role [37].

## CONCLUSION

Video consultations have become part telemedicine in nephrology [38] although uptake remains very variable. As nephrologists are becoming more familiar with this approach, benefits but also limitations are increasingly appreciated (Table 2). Video consultations are not a panacea in providing care but that their main area of use is in providing routine care to stable patients preferably where patient and clinician know each other well. Avoidance of commuting with fossil fuels is an additional advantage. When compared to telephone consultations advantages of video [39] include improved rapport and use of visual clues as part of clinical assessment. Some disadvantages relate to investment and infrastructure, integration into existing her, and reliance on patients' access to equipment and internet. Overall video consultations appear to be safe and popular when used appropriately. We speculate that climate change and the cost of F2F appointments will make video consultations a routine part of renal care within a decade [40]. Further development should focus on integration into EHR as well as accessibility for patients with special needs and support for patients who are less IT literate.

**Table 2:** Ten tips on how to carry out video consultations in nephrology.

Tip 1: Consider reimbursement and think about infrastructure.
Tip 2: Choose a suitable patient population and ensure availability of F2F assessment when required.
Tip 3: Consider technology for home monitoring and wearables.
Tip 4: Consider background audio and camera.
Tip 5: Consider risk and clinical governance.
Tip 6: Think about what is easily missed during a video consultation.
Tip 7: Think about patient experience.
Tip 8: Make video consultations engaging and efficient.
Tip 9: Think about climate change and promote video consultations as an approach to reduce the carbon footprint of care.
Tip 10: Do not assume that everybody is online and support patients who are less IT literate.

**Table 3:** Benefits and limitations with video consultations.

Benefits	Limitations
better than telephone consultations as visual cues can be used by provider	requires investment in infrastructure, software
improved rapport with patient compared to telephone encounter	works best where there is provider continuity (less common overall)
convenient to patients: avoids commute (time commitment and cost)	less IT literate patients may need help from relatives or from provider
avoids conflict with employers and may reduce stigma/reduce time off work	confidentiality issues where patients attend from workplace or public places
probably very cost effective as overheads for F2F clinics avoided	connection: internet is bad in a lot of places (regional Australia at least!)
provider convenience: can be done from home reducing commute and cost	cannot physically assess the patient
does not rely on availability of clinic rooms and staff, can be done *ad hoc*	fear of missing clinical signs nephrology (e.g. rash, murmur, frailty)
triage approach whereby stable whereby F2F if unwell/complex	relies on good internet connection
positive impact on carbon footprint	vulnerable to IT disruption, cyber attack

## Data Availability

No new data were generated or analysed in support of this research.
